# Genetic Differentiation and Selection Signatures Revealed by Two Successive Genomic Selection of Large Yellow Croaker Against Parasite *Cryptocaryon irritans*

**DOI:** 10.1111/eva.70120

**Published:** 2025-06-20

**Authors:** Ji Zhao, Jiaying Wang, Qiaozhen Ke, Junjia Zeng, Yin Li, Zhou Jiang, Fei Pu, Tao Zhou, Ning Li, Peng Xu

**Affiliations:** ^1^ State Key Laboratory of Mariculture Breeding, College of the Environment and Ecology Xiamen University Xiamen China; ^2^ Fujian Key Laboratory of Genetics and Breeding of Marine Organisms, College of Ocean and Earth Sciences Xiamen University Xiamen China; ^3^ Laboratory for Marine Biology and Biotechnology Qingdao Marine Science and Technology Center Qingdao China

**Keywords:** *Cryptocaryon irritans*, genetic differentiation, genomic selection, large yellow croaker, selection signature

## Abstract

The large yellow croaker is one of the most important marine aquaculture species in China, yet its intensive farming industry faces challenges from various pathogens, particularly white spot disease caused by Cryptocaryon irritans. This study aimed to address the issue of white spot disease through genetic breeding. We implemented two consecutive generations of genomic selection (GS) of large yellow croaker against *Cryptocaryon irritans*, resulting in three continuous generations for subsequent analyses. Challenge tests demonstrated significantly higher 96‐h survival rates in the selected generations compared to corresponding controls, with increases of 18.5% and 79.7%, respectively. Survival analysis confirmed that the two selected generations exhibited significantly stronger resistance to *C. irritans* . By merging the genotype files across generations, a comprehensive dataset containing 1844 individuals and 28,637 SNPs was created. Genomic Estimated Breeding Values (GEBVs) showed steady increases across the three consecutive generations, while genetic structure analysis revealed progressive population differentiation resulting from the two rounds of GS. Through genome‐wide selection signature scanning, we identified five positive selection regions (PSRs) distributed across four chromosomes. These regions were enriched for multiple biological pathways related to energy metabolism, immune response, and cell death, including the HIF‐1 signaling pathway, NOD‐like receptor signaling pathway, and apoptosis. Within these pathways, we identified key candidate genes, including crebbp in the HIF‐1 signaling pathway and traf2 involved in immune regulation, both significantly associated with resistance to *C. irritans* . Our results validate the effectiveness of GS in selective breeding of large yellow croaker against *C. irritans* and demonstrate that just two consecutive generations of GS can induce substantial differentiation in genetic structure. This approach facilitates the identification of candidate genes and biological pathways associated with disease resistance.

## 1 Introduction

The large yellow croaker ( *Larimichthys crocea* ) is one of the most economically important marine aquaculture species in China, with a long history of cultural significance in food. Due to overfishing during the 1950s and 1980s, the fishery resources of large yellow croaker have become depleted (Liu and de Mitcheson 2008). Following the breakthrough of artificial reproduction, annual production is now mainly derived from aquaculture. However, the intensive aquaculture industry located within the fjord is often impaired by outbreaks of various pathogenic organisms, leading to significant economic losses. The most severe disease is white spot disease, caused by the ciliate parasite *Cryptocaryon irritans*. In suitable summer temperatures, the life cycle of *C. irritans* is shortened and often results in an overinfection, which eventually leads to fish mortality (Li et al. 2022). Compared with other species, the large yellow croaker is more susceptible to infection by *C. irritans* (Yin et al. 2018). There is an urgent need to alleviate the losses caused by white spot disease in the large yellow croaker farming industry. Given the lack of cost‐effective and labor‐efficient measures for disease prevention and control, utilizing genetic selection to develop new strains of large yellow croaker with enhanced white spot disease resistance represents the most promising solution.

The development of artificial selection has a long‐term history, from domestication in ancient times to modern molecular‐assisted breeding. Throughout the history of selective breeding, the focus has shifted from simple Mendelian traits to more complex quantitative traits. Additionally, the selection of superior individuals has evolved from phenotype‐based methods to breeding value‐based approaches. The estimation of breeding values has also transitioned from being based solely on pedigree information to incorporating extensive molecular marker data. Currently, genomic selection (GS) is one of the most cutting‐edge genetic breeding technologies. GS is a powerful breeding tool that utilizes genomic data to predict the breeding values of individuals based on their genotypes. The method integrates genomic information into the breeding process by estimating the effects of thousands of genetic markers distributed across the genome. These estimates are used to predict the performance of untested individuals, allowing for more accurate and faster genetic improvement. Since its introduction by Dr. Meuwissen Theo (Meuwissen et al. 2001), GS has steadily evolved from a theoretical concept to a practical application, driven by advancements in high‐throughput sequencing technologies. At present, GS has been widely used in the breeding of complex traits of livestock, crops, and aquatic animals, and has obtained remarkable achievements. For instance, genomic selection has revolutionized dairy cattle breeding in the United States. In the period of seven years after the implementation of GS in 2009, rates of genetic gain in Holstein breed per year increased from ∼50% to 100% for yield traits (milk, fat, protein) and from three‐fold to four‐fold for lowly heritable traits (cow fertility, lifespan in the herd, mammary gland health) (García‐Ruiz et al. 2016). Now, producers have accepted genomic evaluations as accurate indications of a bull's potential, replacing traditional daughter‐based evaluation (Wiggans et al. 2017). In terms of GS application in aquatic animals, the reliability of genomic selection was first addressed using cross‐validation in the breeding of Atlantic salmon for sea lice resistance in 2014, and the results revealed that genomic models outperformed the classical pedigree‐based models (Odegård et al. 2014). Subsequently, the theoretical feasibility of GS was also demonstrated in the breeding of other aquatic animals (Houston et al. 2020; Yáñez et al. 2023). It is worth noting that in the breeding of large yellow croaker, Atlantic salmon, and rainbow trout, the researchers have used progeny testing to further confirm the practicability of GS (Vallejo et al. 2016, 2024; Verbyla et al. 2022; Zhao, Bai, et al. 2021). These successful cases have strongly promoted the usage of GS in aquatic animal breeding. Additionally, the strains that were rapidly obtained by using GS will become valuable experimental materials for the genetic analysis of target traits, such as scanning for genome‐wide signatures of selection related to those traits.

Selection signatures are defined as genomic regions that harbor DNA sequences involved in genetic variation of traits subjected to natural or artificial selection (López et al. 2015; Qanbari et al. 2012). Selection signatures can arise as a consequence of the hitchhiking effect, whereby genetic loci linked to a beneficial allele increase in frequency due to their physical proximity to the actual target of selection (Smith and Haigh 2007). However, it is important to recognize that some loci are themselves the direct targets of selection, and their frequency changes are a direct consequence of selection acting on the trait of interest, rather than due to linkage with another selected site. Due to advances in genomic technologies and the reduction of genotyping costs, studies related to genomic scans of selection signatures in aquatic animals have increased in recent years. This trend is motivated by the success of associating genes involved in selection signatures with environmental adaptation or other beneficial traits. For example, research on scanning selection signatures for wild large yellow croaker populations has revealed that genes related to developmental processes were under strong natural selection in the northern population, whereas immune‐related genes were found to be selected in the southern populations (Chen, Bai, et al. 2023). Numerous studies on other aquatic species have also shown that functional genes have been subject to strong selection in the lengthy process of adaptation to diverse environments (Chen, Zhou, et al. 2023; Shen et al. 2019). Since most of the fish species have a short domestication history, many studies have focused on detecting relatively recent selection signatures. For instance, a study searching for signatures of selection in cultured Cermaq Atlantic salmon populations, with a selective breeding history of approximately 50 years, revealed that genes with molecular functions potentially related to growth, response to pathogens and environmental stressors, and early sexual maturation were under strong selection (Gutierrez et al. 2016). Another similar study on farmed rainbow trout also found that candidate genes involved in selection signatures were associated with traits such as growth, early development, reproduction, behavior, and immune system functionality (Cádiz et al. 2021). Limited literature exists on detecting selection signatures in the early stage of artificial selection and investigating the impact of short‐term artificial selection on population structure, even when selection has been implemented for only one or a few generations. Since GS provides higher breeding value estimation accuracy than traditional pedigree‐based methods, it can accelerate genetic improvement. Therefore, it is highly significant to assess the impact of GS by detecting whether short‐term GS can lead to selection signatures and cause genetic differentiation.

Herein, we employed two GS practices to generate two successive generations of *C. irritans* resistant strains in the selective breeding of white spot resistance in the large yellow croaker. We then evaluated fine‐scale population structure divergence across the three successive generations and detected selection signatures across the genome during both GS cycles to investigate the mechanisms underlying white spot resistance. A highlight of our study is that genomic signatures of selection were elucidated through analysis of lineages subjected to accelerated breeding via GS protocols, complementing our conventional Genome‐Wide Association Study (GWAS) approaches. This experimental design enabled the interrogation of the genetic architecture underlying the resistant phenotype through dual methodologies: identifying genomic regions exhibiting selection footprints, while also examining direct associations with phenotypic variance. Our selection‐based methodology provides complementary insights to traditional association studies by capturing loci responsive to artificial selection pressure, potentially revealing functional genetic elements that might be obscured in standard GWAS due to complex genetic interactions or environmental dependencies. This integrated approach offers a more comprehensive understanding of the genetic basis for the target trait than either method alone could provide.

## 2 Materials and Methods

### 2.1 Ethical Approval

The study was approved by the Laboratory Animal Management and Ethics Committee, College of Ocean and Earth Sciences, Xiamen University. All experimental procedures involving large yellow croakers were performed according to the Regulations for the Administration of Affairs Concerning Experimental Animals (College of Ocean and Earth Sciences, Xiamen University).

### 2.2 Genomic Selection Methodology

The procedure for implementing Genomic Selection (GS) is described in detail in our previous study (Zhao, Bai, et al. 2021). In brief, the GS process consists of two main steps. The first step involves establishing a reference population, for which both phenotypic and genotypic data are obtained. The genotype file typically includes thousands of genetic loci, and a mathematical regression relationship between phenotype and genotype is developed to form the genomic selection model. The second step entails creating a selection candidate population, sequencing their genomes to obtain their genotypes, and using the genomic selection model to calculate the genomic estimated breeding values (GEBV) for the candidates. Based on the GEBV, superior individuals can be selected to reproduce the next generation.

In this study, GS was implemented using the BGLR (Bayesian Generalized Linear Regression) package in R. BGLR is a flexible framework for implementing a range of Bayesian models, which are particularly useful for genomic selection in complex traits. Genomic data, typically consisting of SNP genotypes, are coded in a matrix format, where rows correspond to individuals and columns represent SNPs. The phenotype data (trait measurements) for each individual are also organized in a vector format. The basic genomic prediction model is expressed as:

y=Xβ+ϵ

where *y* is the vector of phenotypic values, *X* is the matrix of genotypes, *β* represents the vector of marker effects, and *ϵ* is the vector of residuals. In this study, the Bayesian LASSO model was employed, where priors for the SNP effects are assigned using a Laplace distribution. Using MCMC, the posterior distribution of the marker effects *β* is estimated. BGLR utilizes algorithms such as Gibbs sampling to iteratively update the values of the parameters, including the genetic effects, until convergence is achieved. In this study, 50,000 iterations were set. This iterative process allows the model to compute the posterior mean of the SNPs, which are subsequently used to predict the genetic values of untested individuals. Once the posterior distribution of the SNP effects is obtained, GEBVs for each individual in the selection candidate population can be predicted using the formula:

$$\hat{g}=X\hat{\beta }$$

where $\hat{g}$ represents the predicted GEBVs, and $\hat{\beta }$ are the estimated SNP effects.

### 2.3 The Three Successive Generations of Large Yellow Croaker

Two successive GSs were applied to improve the resistance performance of large yellow croaker against *C. irritans* , thus generating two consecutive resistant strains. These two resistant generations (F2 and F3), together with the first generation (F1), which did not undergo selection for *C. irritans* resistance, resulted in three successive generations for the following analyses. In the present study, the same reference population was used as in the previous GS study (Zhao, Bai, et al. 2021). Briefly, the reference population was produced in January 2018, consisting of the offspring of 500 broodstocks (F0 generation) from the local geographical population in Ningde, China. The artificial infection of *C. irritans* in the reference population was carried out in July 2018 to obtain the resistant phenotype, represented by the death time and survival status. Since the binary trait (survival status) is closer to the disease resistance trait than the quantitative trait (death time), we used survival status in the GS practice. The first GS was implemented in February 2020. The selected candidates came from the same population as the reference population, and these two populations were treated as the F1 generation. A total of 50 individuals with high GEBV were selected from the F1 selection candidates (*n* = 491) to reproduce the next generation, F2, which was theoretically expected to exhibit good performance against *C. irritans* . When the F2 generation reached four months of age, which coincided with the frequent occurrence of white spot disease, a *C. irritans* challenge test was conducted to assess resistant performance. Two years later, we implemented the second GS practice. The reference population, the same as that used in the first GS, was employed to calculate GEBV for F2 selection candidates (*n* = 523). In total, 50 individuals with high GEBV were selected as broodstocks to reproduce the F3 generation. The challenge test for the F3 generation was carried out in June 2022.

### 2.4 Sequencing and Genotyping

The double‐digest restriction‐associated DNA (ddRAD) sequencing approach was used in the first GS. From the F1 reference population, 480 individuals, including 240 deaths and 240 survivors, and 499 individuals in the F1 selection candidates were selected to extract genomic DNA and construct ddRAD libraries, which were sent to Novogene Technology Co. (Tianjin, China) for Illumina HiSeq Paired‐end 150 bp sequencing. Both STACKS v2 and BWA v0.7.17 programs were used to map the clean reads to the reference genome, allowing for the acquisition of the raw genotype. Quality control for SNPs was implemented using the PLINK v1.9 program, with individuals calling rates higher than 0.90, SNP loci calling rates higher than 0.9, and minor allele frequencies higher than 0.05. BEAGLE v5.0 and HAPLOVIEW v4.2 programs were used to impute missing genotypes and find tagging SNPs, respectively. After filtering, a genotype file with 942 individuals and 12,385 SNPs was generated for the first GS practice.

In the second GS practice, the sequencing strategy was updated to chip sequencing, which ensured the stability and consistency of the SNP loci. The same F1 reference population used in the first GS was also treated as the reference population for the second GS. The difference was that the reference population was genotyped with the Large Yellow Croaker 55 K NingXin‐II solid‐phase array (Zhou et al. 2022). The F2 selection candidates were genotyped using the Large Yellow Croaker 55 K NingXin‐III liquid‐phase array, which contains almost the same SNP panel as the NingXin‐II solid‐phase array (Wang et al. 2023). The reason for using the NingXin‐III array is that it is more cost‐effective than the NingXin‐II array. The genotypes of the F1 reference population and F2 selection candidates were merged into one genotype file and followed by quality control, with SNP loci calling rates higher than 0.99 and minor allele frequencies higher than 0.05. A cleaned genotype file containing 1003 individuals and 41,551 SNPs was used in the second GS practice.

A subset of individuals (*n* = 273) in the F2 generation, which had been tested for *C. irritans* challenge, was also genotyped using the NingXin‐II array. A total of 30 individuals randomly sampled from the one‐year‐old F3 generation and F3 selection candidates were genotyped using the NingXin‐III array. The population used in different generations is summarized in the table below (Table 1). Chip genotype data for different generations were merged into one genotype file. The ddRAD data was not included because the chip and ddRAD data had fewer SNP loci in common. After quality control of the chip genotype data, 1844 individuals and 28,637 SNPs were retained for population structure analysis and genomic scans of selection signatures related to the resistant phenotype.

**TABLE 1:** Details of each population used in different generations.

Population	Sequencing method	Number of individuals
F1 reference population	ddRAD/NingXin‐II array	480
F1 selection candidates	ddRAD	491
F2 test	NingXin‐II array	273
F2 selection candidates	NingXin‐III array	523
F3 randomly‐selected	NingXin‐III array	30
F3 selection candidates	NingXin‐III array	566

### 2.5 *C. irritans* Challenge Test

The life cycle of *C. irritans* consists of three main stages: trophont, tomont, and theront. The trophont is the parasitic stage that resides within the host's epithelium, feeding and causing tissue damage. Upon maturation, it detaches and forms a tomont, an encysted reproductive stage in the external environment where multiple theronts are produced. Theronts are the free‐swimming infectious stage responsible for initiating new infections. The procedure for the *C. irritans* challenge is described in our previous GS study (Zhao, Bai, et al. 2021). Briefly, the tomonts formed from the trophonts settling on the host (the large yellow croaker in the present study) were collected from the bottom of the tank. After approximately two days of tomont incubation at 26°C, the theronts were released. Finally, the number of theronts in 10 μL of seawater was estimated by multiple random sampling to perform a quantitative infection. The *C. irritans* challenge was implemented for F2 and F3 generations at approximately four months of age to test whether the offspring exhibit better resistance performance. The commercial strain, which was reproduced and reared during the same period as the F2 and F3 generations, was treated as the control group in the challenge test. Three replicates of the experimental and control groups were used, with each containing 150 individuals placed in a plastic steel tank (volume = 1 m^3^). The challenge dose was determined from pilot trials and set at 2000 and 2500 theronts per fish in the F2 and F3 challenge tests, respectively. The different challenge doses used for different generations may be attributed to various factors, such as the size of experimental fish and the infectivity of theronts. After infection, the observation lasted for 96 h, and the time of death of each individual was recorded. The criterion for determining death was that the experimental fish lost its balance and could not return to a normal swimming state within 10 s after being touched. The survival package in R version 4.2.0 was used to perform survival analysis for the experimental and control groups.

### 2.6 Population Structure Analysis

Basic statistics were calculated, including observed heterozygosity (*H*o), expected heterozygosity (*H*e), and inbreeding coefficients (*F*h and *F*roh) for each generation using the PLINK v1.9 program. The *F*h value is the canonical estimate of genomic F based on excess SNP homozygosity, while the Froh value refers to the proportion of the genome that is in runs of homozygosity. Nucleotide diversity (*π*) on each chromosome was calculated using VCFtools v0.1.17. We employed three methods to reveal the genetic structure of different generations: principal component analysis (PCA), ancestry component analysis, and tree analysis. PCA was performed using the PLINK v1.9 program. The ADMIXTURE v1.3.0 program was used to estimate the genetic ancestry of each sample, and the pophelper package in R v4.2.0 was used to plot the results. We used the VCF‐kit v0.2.6 program to implement tree analysis based on VCF format and the ggtree package in R v4.2.0 to display the results.

### 2.7 Detecting Genomic Signatures of Selection

We utilized various methods to identify positive selection and detect selection signatures during the two successive GSs, including the fixation index (Fst), the fold change in nucleotide diversity (*ω*), and the extent of haplotype homozygosity (Rsb). These indices were estimated on a sliding 100 kb genomic window with a step of 10 kb. The Fst and nucleotide diversity (*π*) values for each sliding window were calculated using VCFtools v0.1.17. The logarithm of the ratio of *π* values for the two populations was calculated as the *ω* index. The Rsb value was calculated for each SNP using the rehh package in R v4.2.0 and then averaged in each sliding window. Two steps were taken to identify positively selected regions (PSRs).

Firstly, the composite selection score (CSS) index was calculated based on Fst, *ω*, Rsb, and *p*‐value following a previously described method (Avalos et al. 2017). The *p*‐value was obtained from the GWAS analysis for the F1 reference population. The procedure of GWAS analysis was conducted according to our previous GWAS study (Zhao, Zhou, et al. 2021). Because the *ω* and Rsb indices were calculated based on two populations, we used the following equations to calculate these two indices based on the three generations in the present study, reflecting the total fluctuation throughout the process of the two genetic selections:

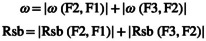

We treated the sliding windows with values exceeding the 99th percentile of the CSS index as preliminary PSRs.

Secondly, four criteria were applied to filter the preliminary PSRs. The first criterion was that $\mathrm{Rsb}\;\left(F2,F1\right)$ and $\mathrm{Rsb}\;\left(F3,F2\right)$ values need to be negative; in other words, the extent of haplotype homozygosity should expand after one selection, because the two genetic selections in this study were directional, and the Rsb index is sensitive to recent positive selection. If the allele frequency of a SNP in survivors was higher/lower than in deaths in the F1 reference population, and correspondingly, the allele frequency of this SNP gradually increased/decreased in the three consecutive generations, then the allelic drift of this SNP was defined as in the right direction. The second criterion was that the number of SNPs with allelic shift in the right direction needed to account for at least one‐quarter of the total number of SNPs in the given PSR. This threshold was set because, through two consecutive generations of selection, the number of SNPs with allelic shift in the right direction was approximately equal to one‐quarter of the total number of SNPs in a given genomic region if that region was not strongly selected. The third criterion was that the *p*‐value and explained genetic variation (EGV) for each window obtained from GWAS analysis in the PSR should be greater than those in its surrounding genomic region. Additionally, the summary statistics of runs of homozygosity (ROH) were implemented using the detectRUNS package in R v4.2.0. The final criterion was that the frequency of ROH should increase with generations. The frequency of ROH refers to the percentage of SNP occurrence in ROH, calculated as the percentage of each SNP falling inside a run in any given population.

**FIGURE 1:**
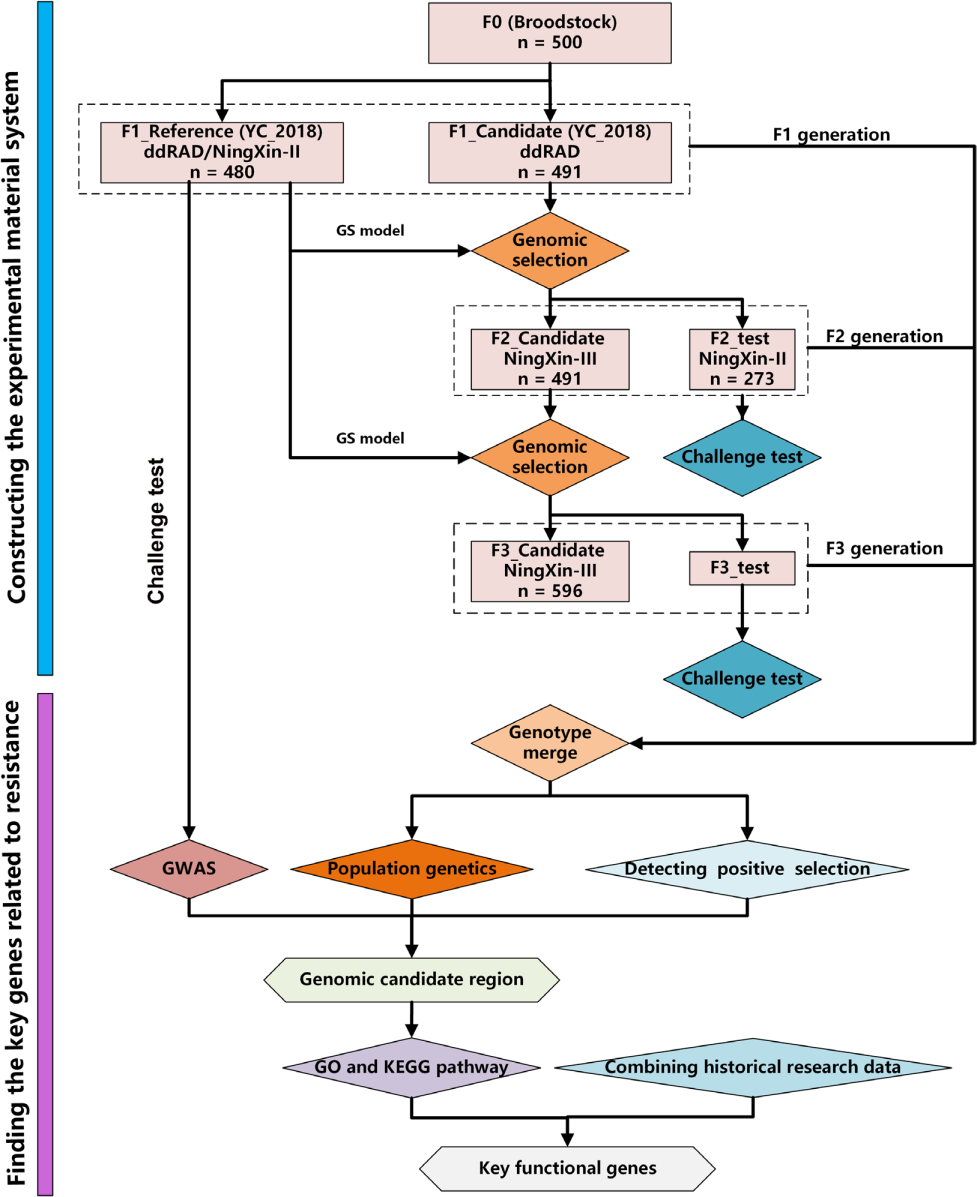
Flowchart of the analysis in the present paper.

After filtering the data based on the above four criteria, a set of PSRs with higher credibility was generated. Gene Ontology and KEGG pathway analyses were performed for each PSR using the clusterProfiler package in R v4.2.0 (Yu et al. 2012). The overall analysis flow of the present study is shown in Figure 1.

## 3 Results

### 3.1 Resistant Performance Against *C. irritans* in F2 and F3 Generations

**FIGURE 2:**
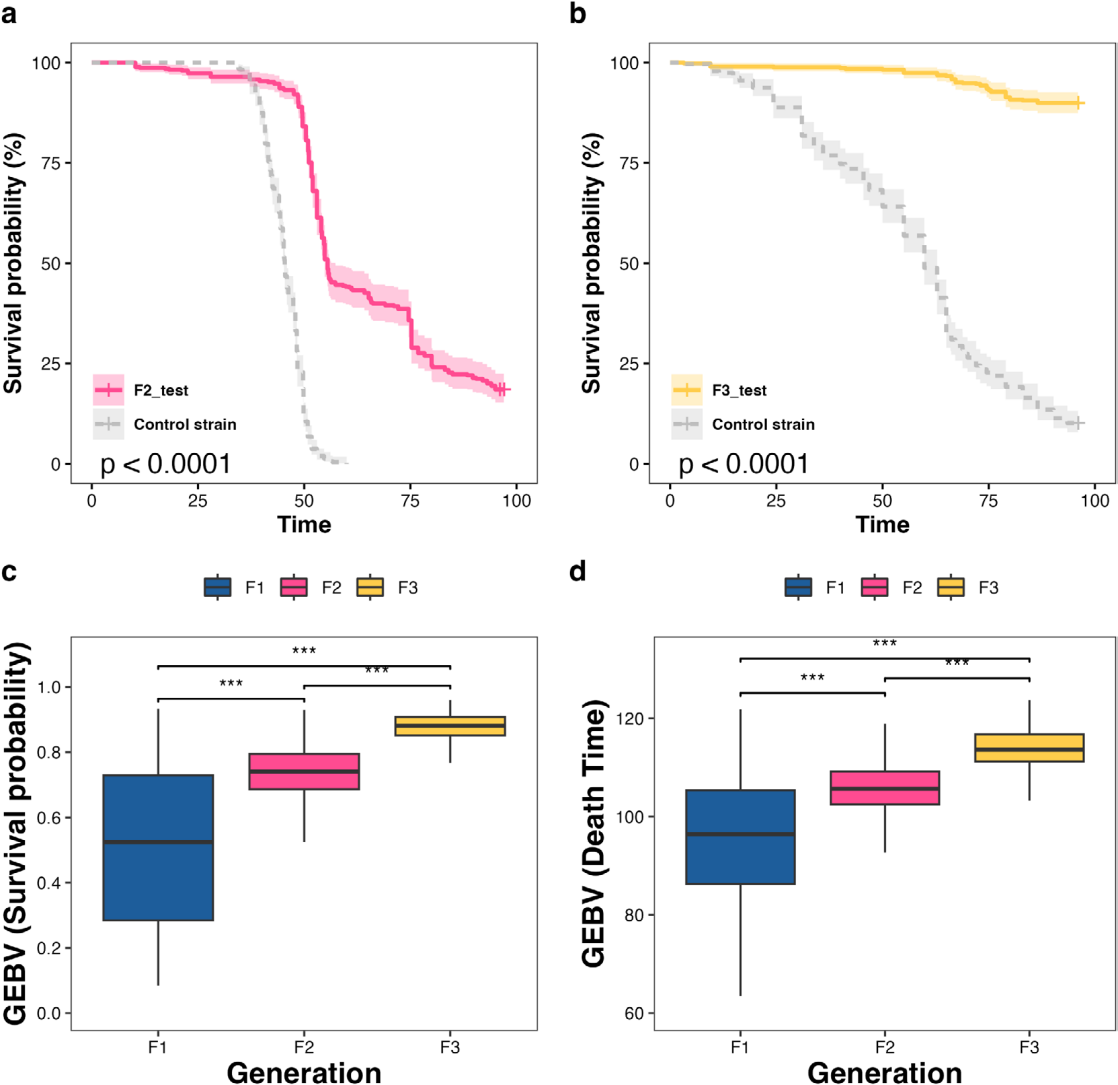
The resistant performance for the F2 and F3 generations that underwent GS for *C. irritans* resistance. (a) The F2 generation exhibited better resistance performance than the control group in the *C. irritans* challenge test. (b) The F3 generation demonstrated superior resistance performance compared to the control group in the *C. irritans* challenge test. (c) When the resistant phenotype was represented as survival status, GEBV (survival probability) steadily increased with the increase of generations. (d) When the resistant phenotype was represented as death time, GEBV (death time) also steadily increased with the increase of generations. The significance level is represented by the number of asterisks: “***” = 0.001 signifies no significance.

In order to confirm the effectiveness of GS, the challenge test of *C. irritans* was conducted on the F2 and F3 generations to detect whether the resistance had been improved. The results of the challenge test for the F2 generation showed that the 96‐h survival rate of the F2 generation was 18.5%, while the control group declined to 0 (Figure 2a). The results of the challenge test for the F3 generation indicated that the 96‐h survival rate was 89.9%, while the control group was only 10.2% (Figure 2b). Survival analysis revealed that compared with the control group, the resistance performance of the F2 and F3 generations was significantly improved, which demonstrated the effectiveness and practicability of GS technology in the genetic selection of complex traits. It is worth noting that due to the susceptibility of the challenge tests to external environmental factors, meaningful comparisons between the challenge results across different generations are unlikely to be feasible. Furthermore, the qualitative significance of the challenge tests outweighs their quantitative significance. Therefore, we calculated the GEBV of the three generations, as GEBV provides a better indication of the resistance improvement in each generation. The results showed that regardless of whether the resistance phenotype was measured by death time or survival status, GEBV increased with each generation (Figure 2c,d), which indirectly demonstrated the effectiveness of the two consecutive generations of GS.

### 3.2 Population Structure Differentiation in the Three Successive Generations

Basic population parameters were estimated to determine the status of each generation (Table 2). For observed heterozygosity (*H*o) and the inbreeding coefficient based on SNP homozygosity (*F*h), there were no significant differences among the three generations, but the inbreeding coefficient based on ROH increased with generations (Figure S1a), which indicated that the inbreeding coefficient showed an increasing trend with continuous genetic breeding. Expected heterozygosity (*H*e) and nucleotide diversity (*π*) both declined with generations (Figure S1b), but did not reach a significant level, which suggested that as selection continued, genetic diversity showed a decreasing trend.

**TABLE 2:** Basic population parameters for the three generations.

Generation	*H*o	*H*e	*F*h	*F*roh	*π*
F1	0.288	0.300	0.025	0.0534	8.969E−06
F2	0.296	0.292	−0.003	0.0587	8.719E−06
F3	0.293	0.277	0.006	0.0748	8.278E−06

**FIGURE 3:**
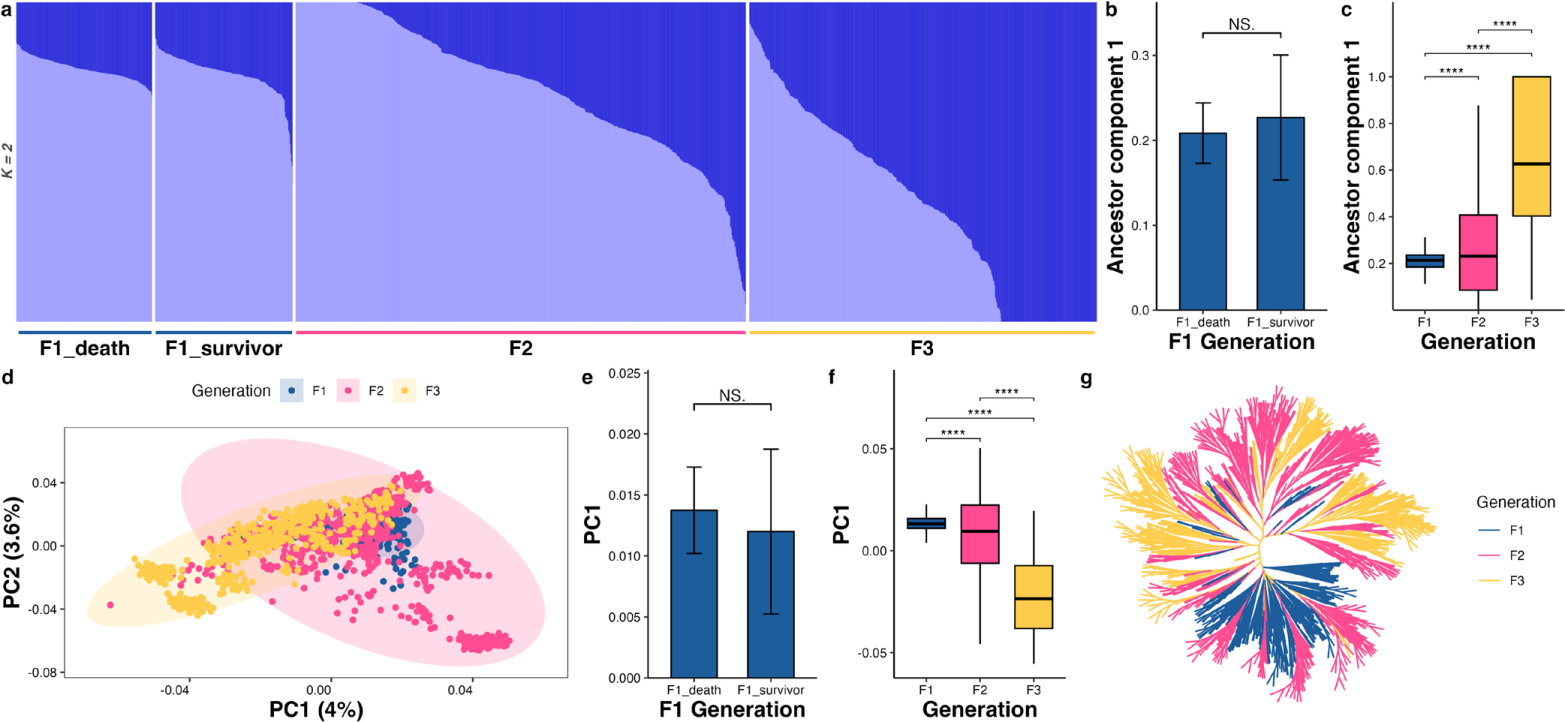
Genetic structure analysis revealed that the population structure among the three consecutive generations gradually differentiated with the ongoing two GSs. (a) Ancestral admixture among the three consecutive generations, with the number of populations set to 2. (b) The mean value of ancestor component 1 in the survivors of the F1 generation was higher than in the deceased individuals, but did not reach a significant level. (c) With the increase of generations, the mean value of the ancestor component 1 gradually increased, indicating the occurrence of genetic divergence. (d) Principal component analysis (PCA) revealed that the population structure gradually shifted to the left with the increase of generations. (e) The mean value of PC1 in the survivors of the F1 generation was lower than the deceased individuals, but did not reach a significant level. (f) With the increase of generations, the mean value of PC1 gradually decreased. (g) The result of the tree analysis was consistent with the results of PCA and ancestor component analysis. The significance level is represented by the number of asterisks: “****” = 0.0001 and “NS” signifies no significance.

Then the genetic structure of each generation was investigated via multiple methods. The two‐dimensional PCA score plot revealed that there was no significant independent grouping structure among the three generations (Figure 3d). However, we found that as the generation increased, the group structure gradually shifted to the left; in other words, the average value of the first principal component (PC1) gradually decreased (Figure 3e), and the average PC1 value of survivors in the F1 reference population was lower than that of dead individuals (Figure 3f), which indicated that the selection was heading in the expected and desirable direction. Ancestry component analysis also confirmed the above results. When *K* = 2, the average value of the ancestor component 1 increased with generation (Figure 3a), and the average value of this ancestor component 1 of survivors in the F1 reference population was higher than that of dead individuals (Figure 3b). A phylogenetic tree also supported the clear population structure, coinciding with the results of PCA and ancestry component analysis (Figure 3g). The F1 and F3 generations had relatively independent topological structures in the tree plot, and the F2 generation group was mixed in between them. In summary, the genetic structure of the three successive generations clearly revealed the existence and direction of artificial selection and demonstrated the power of GS.

### 3.3 Positively Selected Regions Under the Two Successive GSs

**FIGURE 4:**
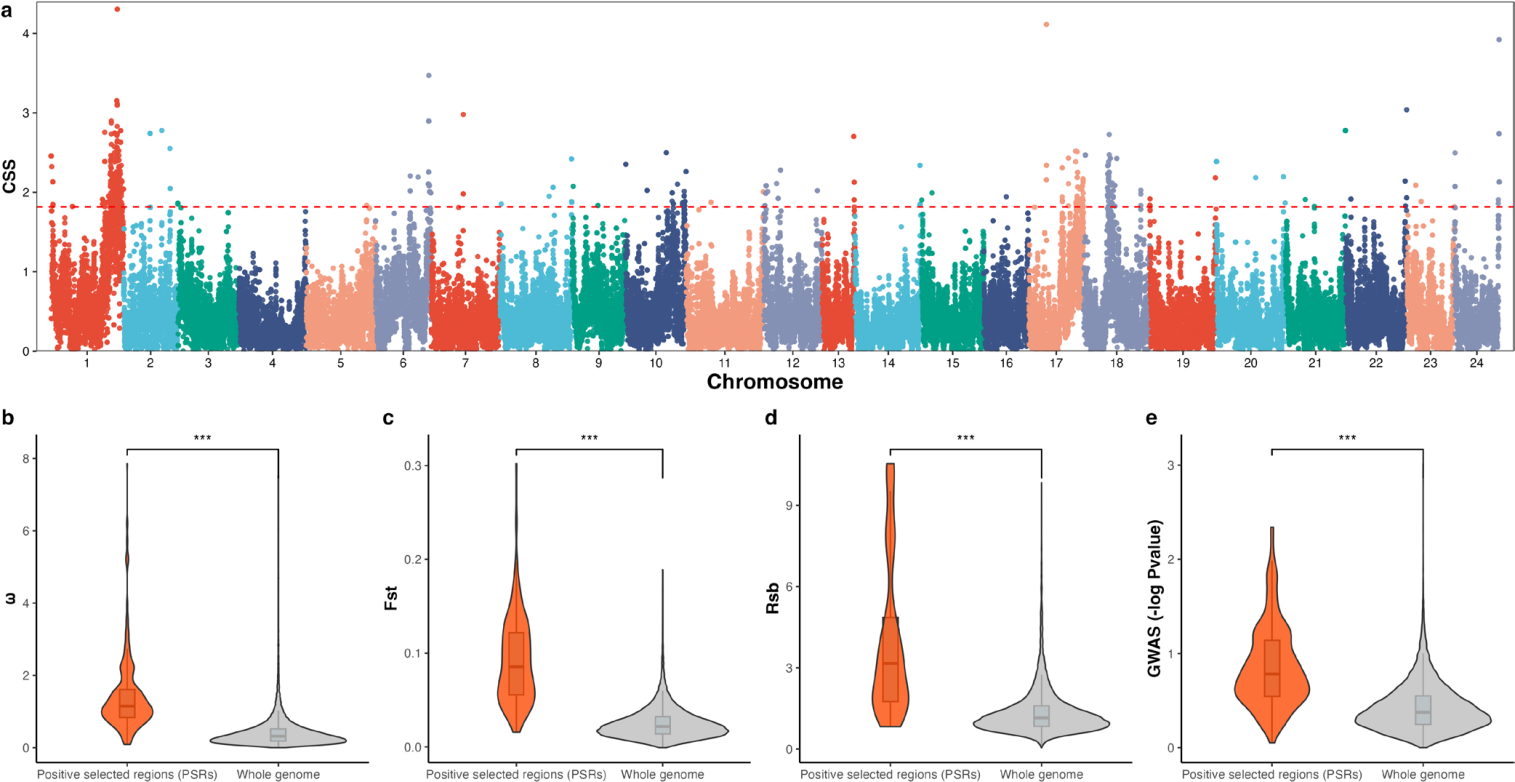
Scanning whole‐genome selection signatures using the composite selection score (CSS) index. CSS was calculated based on Fst, *ω*, Rsb, and *p*‐value following a previously described method. (a) The Manhattan plot of the CSS index. The genomic region exceeding the red threshold line was treated as a preliminary positive selection region (PSR). (b) The mean value of *ω* in preliminary PSRs was significantly higher than that in the whole genome. (c) The mean value of Fst in preliminary PSRs was significantly higher than that in the whole genome. (d) The mean value of Rsb in preliminary PSRs was significantly higher than that in the whole genome. (e) The mean value of *p*‐value obtained from GWAS results in preliminary PSRs was significantly higher than that in the whole genome. The results indicate that CSS is a better index for identifying selection signatures. The significance level is represented by the number of asterisks: “***” = 0.001 signifies no significance.

**FIGURE 5:**
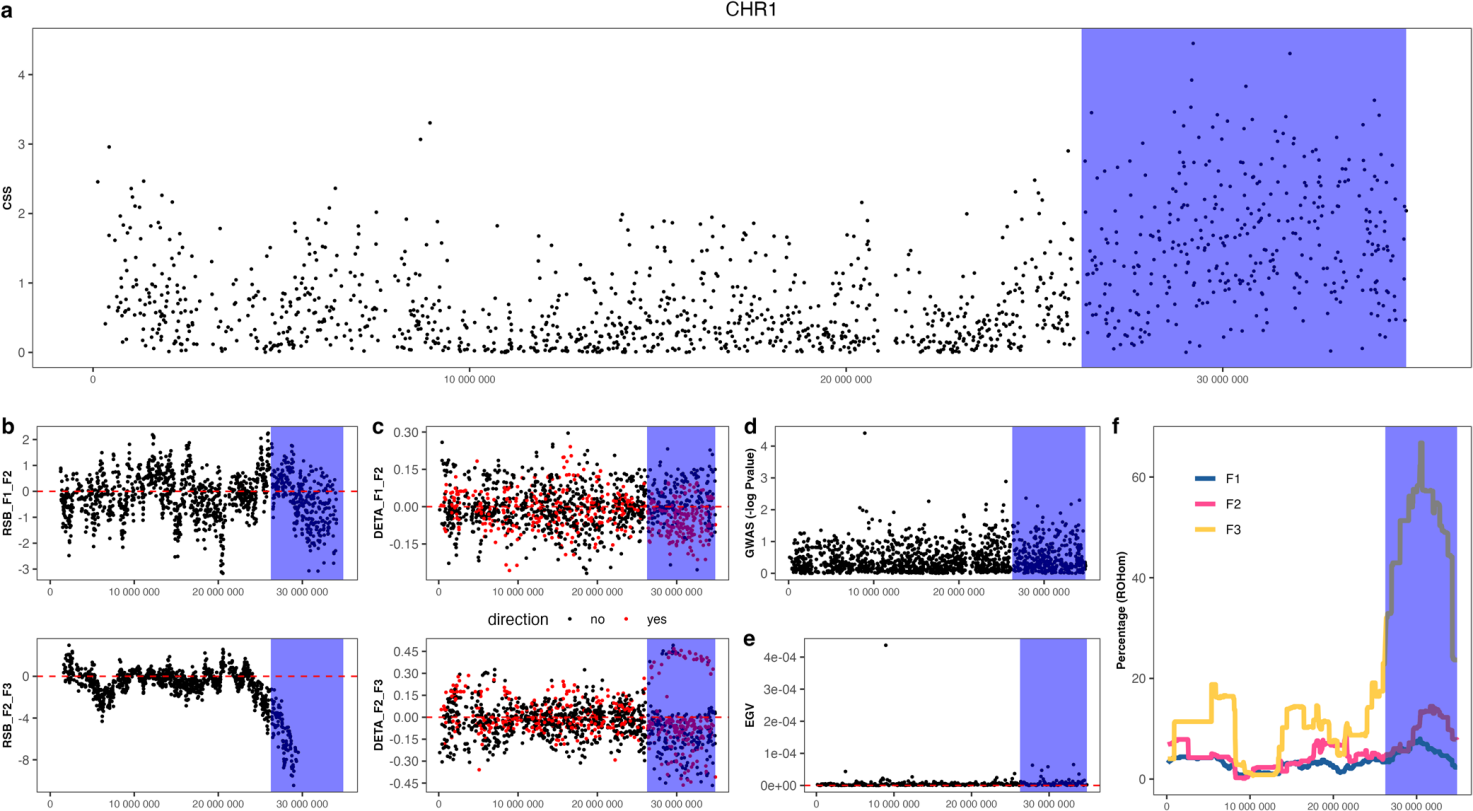
Detailed information of the PSR on chromosome 1. (a) Genomic landscape of selection signatures represented by CSS in chromosome 1. The colored shadow area indicates the PSR. (b) The top plot represents the Rsb index calculated based on the F1 and F2 generations. The bottom plot represents the Rsb index calculated based on the F2 and F3 generations. It can be observed that during the two GSs, the extent of haplotype homozygosity in the PSR expanded (Rsb < 0). (c) The allelic shift in the process of two GSs. Red dots represent the SNPs with allelic shift in the right direction, while black dots represent SNPs in the wrong direction. (d, e) From the top and bottom plots, respectively, the GWAS *p*‐value and EGV (explained genetic variation) in the PSR were examined, showing higher values than the surrounding region. (f) The frequency of ROH (runs of homozygosity) in the PSR increased with the increase of the generations, further confirming that this long PSR underwent strong selection.

GWAS analysis in the F1 reference population was implemented, and three indices (*ω*, Fst, and Rsb) were used for scanning genomic selection signatures (Figure S2). We then used the CSS value integrated from the above four indices to screen for preliminary PSRs (Figure 4a). A total of 19 preliminary PSRs were detected, and these PSRs were found to be distributed on 11 chromosomes (Table S1). The average value of the four independent indices in the preliminary PSRs was significantly higher than in the whole genome (Figure 4b–e), which indicated that the CSS index possessed better representation for detecting selection signatures. After further filtering based on four criteria, five PSRs located on chromosomes 1, 10, 17, and 18 were retained (Table 3). In the two GSs, the extent of haplotype homozygosity in the PSR on chromosome 1 expanded (Figure 5b). In this PSR, the number of SNPs with allelic shift in the desired direction (*n* = 188) greatly exceeded one quarter of all SNPs (Figure 5c). Moreover, the GWAS *p*‐value and EGV in this PSR were higher than in its surrounding region (Figure 5d,e). Additionally, the frequency of ROH increased with generation, which indicated that this PSR was under strong selection (Figure 5f). The information of other PSRs is similar to the above description, which is provided in the supplementary plots (Figures S3–, S5).

**TABLE 3:** The details of the final PSRs were obtained through four criteria.

Chr	Bin_start	Bin_end	N_variants	N_right_direction
1	26,250,001	34,872,296	367	188
10	19,770,001	21,420,000	83	48
17	14,680,001	16,850,000	102	47
17	21,580,001	24,960,000	166	87
18	13,170,001	17,440,000	208	107

### 3.4 Functional Pathways and Genes in the Positively Selected Regions Related to Resistance Against *C. irritans*

**FIGURE 6:**
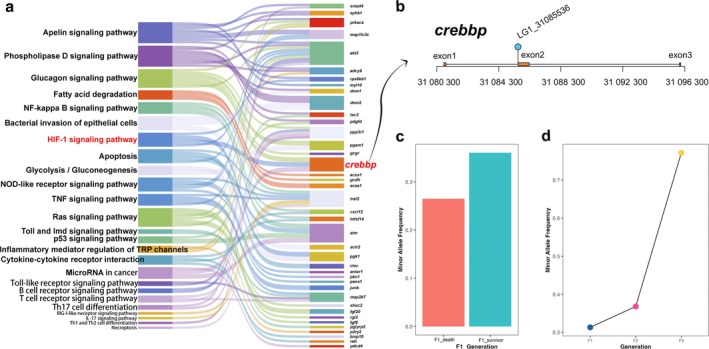
Annotation of PSRs. (a) Important KEGG pathways and genes involved in the PSRs. The width of the box represents the number of genes involved in a pathway or the number of pathways a gene is classified into. (b) The gene structure of *crebbp* which regulates the expression of downstream genes involved in the HIF‐1 signaling pathway. *crebbp* was identified in the PSR on chromosome 1, and one SNP (LG1_31085536) is located in the first intron of this gene. (c) The minor allele frequency of the SNP located in *crebbp* gene in the survivors of the F1 reference population was higher than that in the deceased individuals. (d) With the increase of generations, the minor allele frequency of this SNP rapidly increased. The minor allele in the F1 generation has become a major allele in the F3 generation, indicating that the *crebbp* gene was positively selected and likely related to *C. irritans* resistance.

A total of 750 genes were identified in the PSRs. GO and KEGG enrichment analyses were performed for these genes. All the annotated GO terms and KEGG pathways are listed in the supplementary tables (Tables S2 and S3), and the top 20 GO terms and KEGG pathways with high enrichment factors are provided in the supplementary figures (Figures S6 and S7). Combining the previously published transcriptomics and GWAS data on *C. irritans* infection in the large yellow croaker, numerous biological pathways involved in the molecular response to *C. irritans* infection were also identified in the PSRs detected in the present study. These important biological pathways are related to energy metabolism and immune response (Figure 6a). Biological pathways related to energy metabolism included the “Apelin signaling pathway,” “Phospholipase D signaling pathway,” “Glucagon signaling pathway,” “Fatty acid degradation,” “HIF‐1 signaling pathway,” and others. Biological pathways related to immune response included the “NOD‐like receptor signaling pathway,” “Inflammatory mediator regulation of TRP channels,” and “B cell receptor signaling pathway.” There were also pathways linked to cell death, such as “Autophagy” and “Apoptosis.”

In the previous studies on the molecular response of large yellow croaker against *C. irritans* infection, the HIF‐1 signaling pathway has often been highlighted (Bai, Wang, Zhao, Bai, et al. 2022; Zhang et al. 2020), because the parasitism of theronts destroys the gill tissue, causing a hypoxic state, and thereby inducing the response of the HIF‐1 signaling pathway. In the present study, we found that *crebbp* (Creb‐binding protein, Creb is cAMP response element‐binding protein), a gene involved in the HIF‐1 signaling pathway, was under strong selection. The gene *crebbp* was located in the PSR on chromosome 1. There was a SNP (LG1_31085536) in the first intron of *crebbp* (Figure 6b). The allelic frequency of this SNP in the survivors and deaths in the F1 reference population was respectively 0.361 and 0.265 (Figure 6c), and it changed to 0.368 in the F2 generation, and finally increased to 0.772 in the F3 generation (Figure 6d), which indicated that this SNP tended to become fixed within three generations. In addition, another key candidate gene, *traf2*, was identified in the PSR on chromosome 17; however, no SNP was found to be located in the gene region for observation of allele frequency change. *traf2* is involved in many immune‐related pathways, such as the IL‐17 signaling pathway and NOD‐like receptor signaling pathway. It also plays an important role in the process of apoptosis.

## 4 Discussion

### 4.1 Effectiveness of GS in Selective Breeding of Large Yellow Croaker Against *C. irritans*

By employing the ddRAD sequencing strategy, we have previously confirmed the effectiveness of GS in the selective breeding of large yellow croaker against *C. irritans* (Zhao, Bai, et al. 2021). In the present study, two successive GSs were implemented for the same resistant trait. Compared to the previous GS study, a different base population and genotyping method were employed. Through the challenge test, both the F2 and F3 generations exhibited better resistance performance than the control group. Repeated GS practices have once again proven their superiority in the breeding of a resistant strain of large yellow croaker against *C. irritans* . Compared with livestock and crops, the application of GS in aquatic animals started relatively late, and most studies were limited to using cross‐validation to calculate the accuracy of GS, thereby demonstrating that a trait is suitable for GS breeding. The most direct evidence to prove the validity of GS is through progeny testing of selection candidates. There are currently two common ways to conduct progeny testing of selection candidates. One is suitable for a species where a large number of families can be constructed. For a given target trait, the correlation of the mid‐parent GEBV with the mean progeny performance for each progeny testing family is used to evaluate the predictability of the GS model. Dr. Roger L. Vallejo has conducted many representative research works, such as GS for bacterial cold water disease resistance in rainbow trout (Vallejo et al. 2016, 2017) and sea lice resistance in the aquaculture strain of North American Atlantic salmon (Vallejo et al. 2024). These works have strongly validated the effectiveness of GS in the selective breeding of complex disease‐resistant traits in aquatic animals. Another type of progeny testing of selection candidates is more suitable for species where a large number of families cannot be established, which measures the mean performance gain of the offspring from selection candidates with high GEBV relative to the offspring of the unselected population. In view of this measure, our team has conducted many pioneering and representative research studies on the selective breeding of complex traits in large yellow croaker, such as white spot resistance (Zhao, Bai, et al. 2021), visceral white‐nodules resistance (Bai, Wang, Zhao, Ke, et al. 2022), superior swimming performance (Zeng et al. 2023), and efficient plant protein utilization (Ke et al. 2022). In conclusion, we have repeatedly demonstrated the efficiency and practicality of GS in the selective breeding of large yellow croaker.

### 4.2 GS Rapidly Drives the Divergence of the Genetic Structure Through Three Successive Generations

In the present study, GEBV was examined to gradually increase with generation, and the genetic diversity showed a slight decline. The inbreeding coefficient (*F*roh) gradually increased with generation. Using an ultra‐large US dairy cattle data set that contained 154,008 bulls and 33,022,242 cows born since 1975, researchers have examined the genetic gain (measured as predicted breeding value, PBV) of production traits, such as milk, fat, and protein (Guinan et al. 2023). The results of the above research showed that the PBV of production traits has increased steadily over time, particularly in Holstein and Jersey breeds, which have benefited most from GS, with up to a 192% increase in genetic gain since GS was applied in 2009. A negative impact was that genomic inbreeding was increasing in Holstein bulls and cows, which was consistent with our results. It is widely accepted that long‐term artificial selection can cause the loss of genetic diversity. However, if the selection duration is short, many studies have shown inconsistent results. In a mass selective breeding program for Kumamoto oyster, the F4 generation exhibited significantly lower genetic variation compared to F0 (Ma et al. 2023). The decrease of genetic diversity was observed in the process of three successive mass selections for growth traits in Pacific abalone (N. Chen et al. 2017); however, another independent research study found that the genetic variation over three generations was maintained in the black shell strain of Pacific abalone, which was attributed to using a balanced sex ratio and large size of broodstock (Xu et al. 2019). Compared with those of the wild populations, the abundant genetic diversity of selected lines in *Crassostrea nippona* showed no detectable loss during three generations of mass selection (Hu et al. 2022). So far, there have been no reports on the changes in the genetic diversity of selected strains under GS. Our results showed that nucleotide diversity experienced a decline through the two successive GSs but did not reach a significant level. In the future of continuous selection for white spot resistance, more strategies should be adopted to control the loss of genetic diversity and the increase of the inbreeding coefficient, such as expanding the scale of broodstock and avoiding mating between close relatives.

It is well known that domestication or long‐term artificial selection for a target trait will lead to the divergence of population genetic structure. Most studies focused on the final status of population structure after long‐term selection, but few studies investigated whether genetic structure alters in the initial stages of selection or how genetic structure shifts from generation to generation by analyzing historical genotype data. In the present study, genetic structure analysis revealed that genetic differentiation occurred among the three successive generations. The results from the two studies above did not find significant genetic differentiation in three generations of mass selection for growth traits in Pacific abalone (Xu et al. 2019) and Crassostrea nippona (Hu et al. 2022). No detectable genetic differentiation could likely be due to a very limited number of genetic markers and experimental individuals, which may not have been sufficiently powerful to evaluate genetic differentiation. In contrast, a large number of SNP markers and experimental individuals were included in the present study, ensuring that even subtle genetic differentiation could be detected. Most importantly, we found that the direction of genetic structure shift in the two successive GSs was consistent, and the direction of change was what we expected, moving towards the survivors of the F1 generation. Our results indicated that the utilization of GS can cause detectable genetic structure shifts in just a few generations.

### 4.3 HIF‐1 Signaling Pathway Involved in the Successive Selection for White Spot Resistance in Large Yellow Croaker

We have revealed that significant and gradual genetic differentiation occurred in the two successive GSs, suggesting that artificial selection would theoretically result in genomic selection signatures. Based on the theory of positive selection, many PSRs were identified through the integration of multiple information and comprehensive analyses, and the biological pathways and genes involved in the PSRs would be more likely to be linked to white spot resistance. The HIF‐1 (hypoxia‐inducible factor 1) signaling pathway was enriched in the retained PSRs after filtering based on four different criteria. The primary transcriptional response to hypoxic stress is mediated by the HIF‐1 signaling pathway (Majmundar et al. 2010). The theronts of *C. irritans* often parasitize the gill tissue of the host, which causes damage to the integrity of the gill filament structure, thereby affecting oxygen intake and causing an imbalance in oxygen homeostasis. Therefore, the HIF‐1 signaling pathway is induced to maintain oxygen homeostasis and regulate the downstream metabolic‐related pathways to produce more energy necessary for basic functions. In our previous GWAS of large yellow croaker against *C. irritans* , the HIF‐1 signaling pathway was also annotated in the detected QTL regions (Zhao, Zhou, et al. 2021). A previous transcriptome analysis of large yellow croaker in response to *C. irritans* infection also found that the HIF‐1 signaling pathway was enriched in the gills (Bai, Wang, Zhao, Bai, et al. 2022). Overall, the HIF‐1 signaling pathway may play a vital role in *C. irritans* resistance in the large yellow croaker. As the core gene in the HIF‐1 signaling pathway, the HIF‐1 family was not found in the PSRs. However, another important gene, *crebbp*, was detected in the PSR on chromosome 1, which has been confirmed as a key component involved in the HIF‐1 signaling pathway. Crebbp has intrinsic acetyltransferase functions and is ubiquitous in vertebrates. Its rich binding domains allow it to participate in many regulatory networks and regulate the expression of thousands of genes, thereby maintaining cell homeostasis and assisting in coping with changes in the external environment (Sheikh and Akhtar 2019). Under hypoxic conditions, accumulated Hif‐1α proteins will first recruit Crebbp through its C‐TAD domain, then form a dimer with Hif‐1β through the bHLH domain to become a functional transcription complex. Finally, it binds to the hypoxia response elements (HREs) on the target gene to ultimately initiate the expression (Zimna and Kurpisz 2015). A SNP marker was found to fall into the intron of *crebbp*, and the allelic frequency of this SNP rapidly increased with each generation, suggesting that *crebbp* was under strong selection. Collectively, *crebbp* is an important candidate gene related to *C. irritans* resistance, and further molecular functional validation is needed in the future.

### 4.4 Other Important Biological Pathways Involved in the Successive Selection for White Spot Resistance in Large Yellow Croaker

While the HIF‐1 signaling pathway received particular attention due to its established role in hypoxia response during gill parasitism, several other important pathways warrant discussion as they likely contribute to the complex resistance phenotype. The energy metabolism pathways identified in the PSRs, including Apelin, Phospholipase D, and Glucagon signaling pathways, suggest that modulation of energy homeostasis is crucial for host survival during infection. The Apelin signaling pathway regulates cardiovascular function and energy metabolism (Chng et al. 2013), and its selection may reflect adaptation to the cardiovascular stress induced by gill damage. Similarly, the Glucagon signaling pathway, which regulates glucose metabolism during fasting or stress conditions (Jiang and Zhang 2003), could help maintain energy supplies during the increased metabolic demands of immune response activation. Selection in these pathways suggests that efficient energy utilization and distribution may be as important for survival as direct immune responses. Regarding immune‐related pathways, the identification of the NOD‐like receptor signaling pathway in PSRs is particularly noteworthy. NOD‐like receptors function as intracellular sensors that detect pathogen‐associated molecular patterns and damage‐associated molecular patterns, initiating inflammatory responses through activation of NF‐κB and MAPK signaling (Motta et al. 2015). The selection pressure on this pathway suggests that enhanced pathogen recognition and appropriate inflammatory regulation may be key components of resistance to *C. irritans* . Additionally, the B cell receptor signaling pathway's presence in PSRs indicates that adaptive immunity development may play a role in resistance, potentially through improved antibody production against the parasite (Xu et al. 2016). The selection of signatures in cell death pathways, such as Autophagy and Apoptosis, highlights the importance of controlled cell death in the host response. During *C. irritans* infection, these pathways likely serve dual purposes: elimination of infected or damaged cells and regulation of inflammation (Mariño et al. 2014). The balance between apoptosis and autophagy in infected tissues may determine whether the host can contain the infection while maintaining sufficient tissue function.

Beyond the specific pathways, our identification of TRAF2 under strong selection pressure provides insight into the molecular mechanisms of resistance. TRAF2 is a critical adaptor protein in TNF receptor signaling that regulates NF‐κB activation, JNK signaling, and cell survival decisions (Wajant and Scheurich 2001). Its selection suggests that fine‐tuning of inflammatory responses and cell death regulation may be under evolutionary pressure during artificial selection for resistance. Recent studies in fish have demonstrated that TRAF2 modulates antiviral responses and inflammation (Eslamloo et al. 2017), and its selection in our study suggests similar importance in antiparasitic defense. The identification of these diverse pathways under selection suggests that resistance to *C. irritans* infection involves a complex interplay of energy metabolism, immune activation, and cell death regulation. This multifaceted genetic architecture aligns with previous transcriptomic studies showing broad physiological responses to infection (Zhang et al. 2018) and explains why breeding for resistance has been challenging but achievable through genomic selection approaches that capture these complex genetic interactions.

## 5 Conclusion

In an effort to cultivate a resistant strain to alleviate the economic loss caused by severe white spot disease, we employed two successive GSs to produce three consecutive generations. The significantly improved survival rates observed in the F2 and F3 generations following *C. irritans* challenge tests strongly support the effectiveness and applicability of GS in enhancing resistance traits. Genetic structure analysis indicated that only two successive generations of GS were sufficient to induce substantial genetic divergence within the population. Furthermore, a genome‐wide scan for selection signatures identified several candidate regions potentially subjected to artificial selection. Among these, crebbp, a gene involved in the HIF‐1 signaling pathway, emerged as a key candidate associated with resistance to *C. irritans* . In addition to the HIF pathway, we also highlighted other enriched biological pathways related to immune response, energy metabolism, and cell death, reflecting the complex polygenic architecture underlying resistance. These findings collectively provide new insights into the genetic mechanisms driving disease resistance and demonstrate the power of GS as a practical tool for enhancing disease resistance in aquaculture species.

## Ethics Statement

This study was approved by the Animal Care and Use Committee at the College of Ocean and Earth Sciences, Xiamen University. All the methods used in this study were carried out in accordance with approved guidelines.

## Conflicts of Interest

The authors declare no conflicts of interest.

## Supporting information


**FIGURE S1.** Variation of genomic inbreeding coefficient and nucleotide diversity among the three consecutive generations. (a) Genomic inbreeding coefficient (*F*roh) based on runs of homozygosity (ROH) gradually increased during the two continuous GSs. (b) With the progression of two GSs, nucleotide diversity (*π*) showed a downward trend but did not reach a significant level. The significant level is represented by the number of asterisks: “***” = 0.001, “**” = 0.01, “*” = 0.05, and “NS.” signifies no significance.

**FIGURE S2.** Manhattan plot of GWAS result and genomic selection signatures using various methods. (a) GWAS result based on the F1 reference population. (b) Scanning whole‐genome selection signatures using the index of the fold change in nucleotide diversity (*ω*). (c) Scanning whole‐genome selection signatures using the index of the fixation index (Fst). (d) Scanning whole‐genome selection signatures using the index of the extent of haplotype homozygosity (Rsb).

**FIGURE S3.** Detailed PSR on chromosome 10. The description of each plot is similar to the PSR on chromosome 1 described in the main body.

**FIGURE S4.** Detailed of PSR on chromosome 17.

**FIGURE S5.** Detailed of PSR on chromosome 18.

**FIGURE S6.** The top 20 enriched GO terms for all of genes involved in the PSRs.

**FIGURE S7.** The top 20 enriched KEGG pathways for all of genes involved in the PSRs.

**TABLE S1.** Preliminary PSRs identified through CSS filtering.

**TABLE S2.** Detailed information on the GO terms that all genes involved in the PSRs were classified into.

**TABLE S3.** Detailed information on KEGG pathways that all genes involved in the PSRs were classified into.

## Data Availability

The raw data and running scripts utilized in this study will be made available upon request, if deemed necessary.
