# Supplementary figures and images for: Genetic Differentiation and Selection Signatures Revealed by Two Successive Genomic Selection of Large Yellow Croaker Against Parasite Cryptocaryon irritans
Source: Evol Appl. 2025 Jun 20;18(6):e70120. doi: 10.1111/eva.70120 (PMC12181394; doi:10.1111/eva.70120)

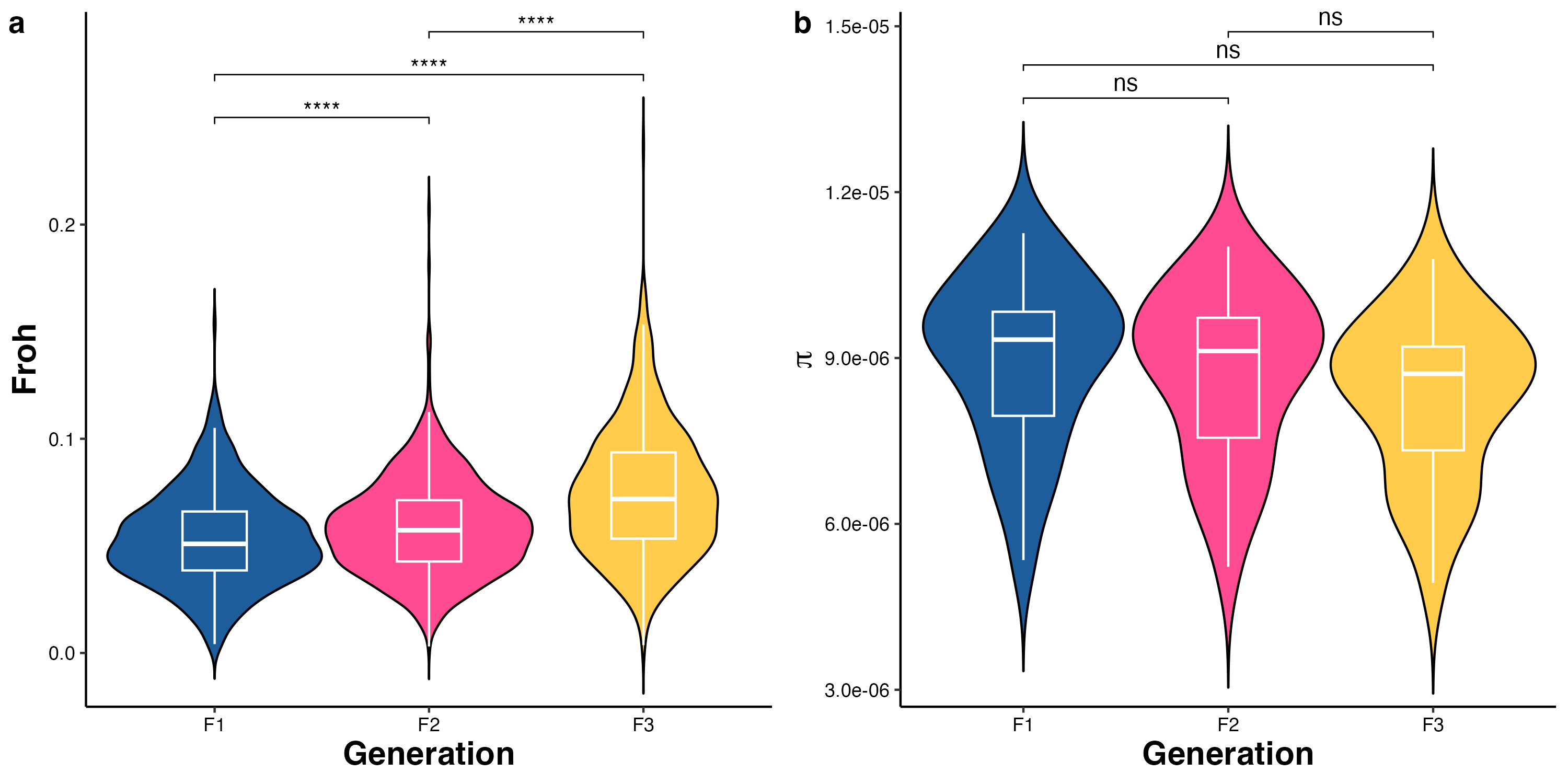

Supplement: Supplementary file 1 — FIGURE S1. Variation of genomic inbreeding coefficient and nucleotide diversity among the three consecutive generations. (a) Genomic inbreeding coefficient (Froh) based on runs of homozygosity (ROH) gradually increased during the two continuous GSs. (b) With the progression of two GSs, nucleotide diversity (π) showed a downward trend but did not reach a significant level. The significant level is represented by the number of asterisks: “***” = 0.001, “**” = 0.01, “*” = 0.05, and “NS.” signifies no significance. [file EVA-18-e70120-s010.png]

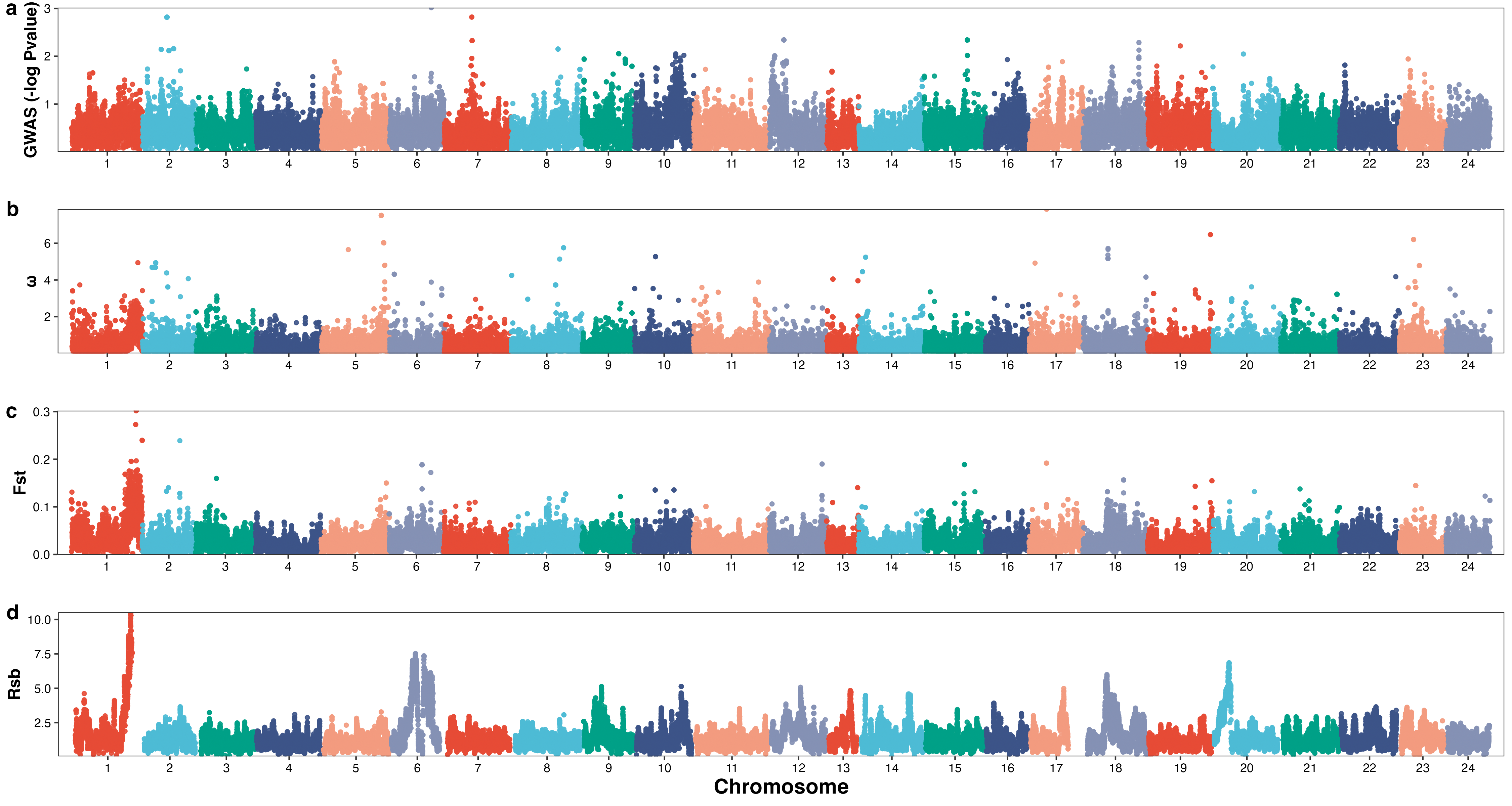

Supplement: Supplementary file 2 — FIGURE S2. Manhattan plot of GWAS result and genomic selection signatures using various methods. (a) GWAS result based on the F1 reference population. (b) Scanning whole‐genome selection signatures using the index of the fold change in nucleotide diversity (ω). (c) Scanning whole‐genome selection signatures using the index of the fixation index (Fst). (d) Scanning whole‐genome selection signatures using the index of the extent of haplotype homozygosity (Rsb). [file EVA-18-e70120-s006.png]

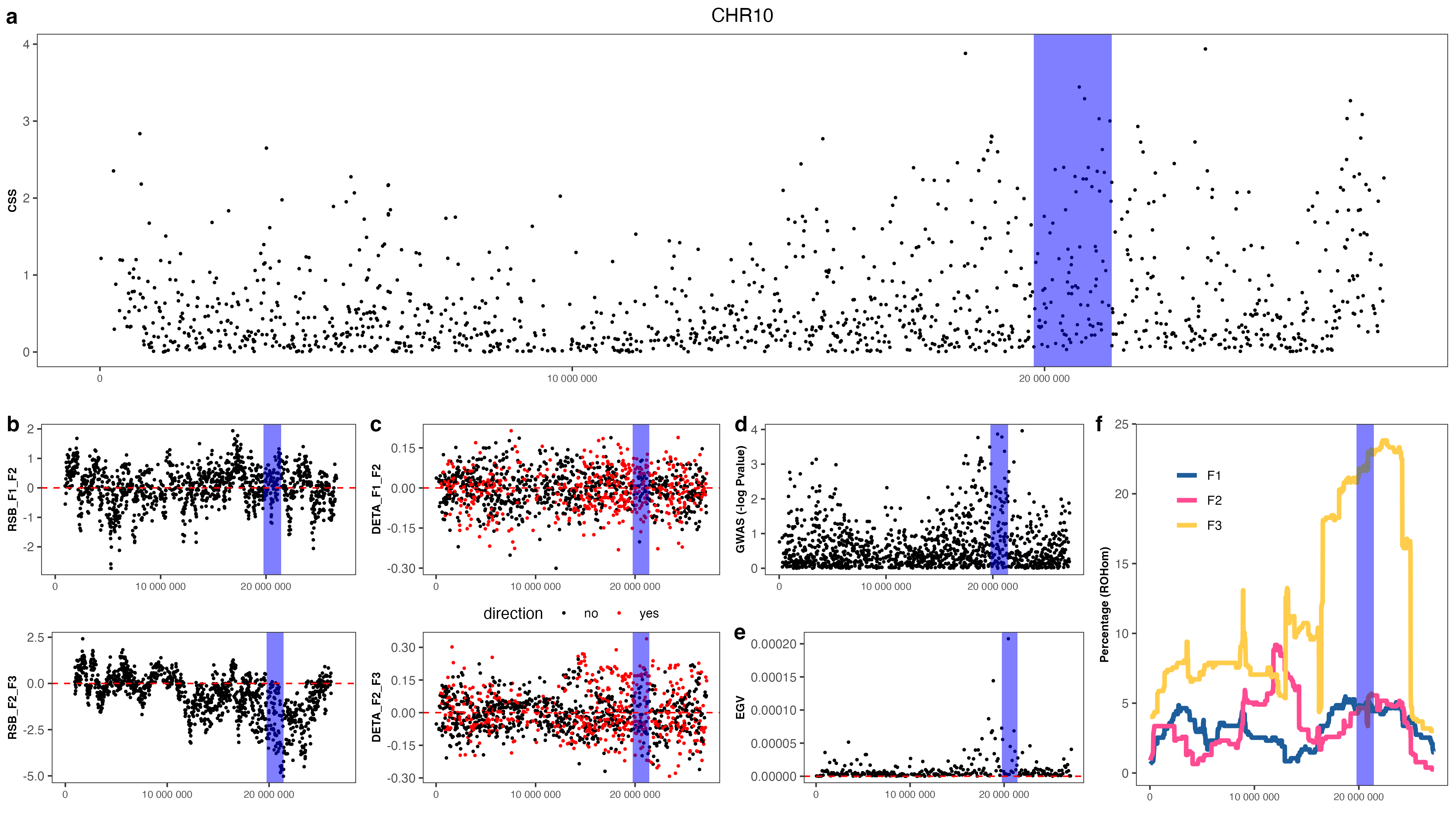

Supplement: Supplementary file 3 — FIGURE S3. Detailed PSR on chromosome 10. The description of each plot is similar to the PSR on chromosome 1 described in the main body. [file EVA-18-e70120-s001.png]

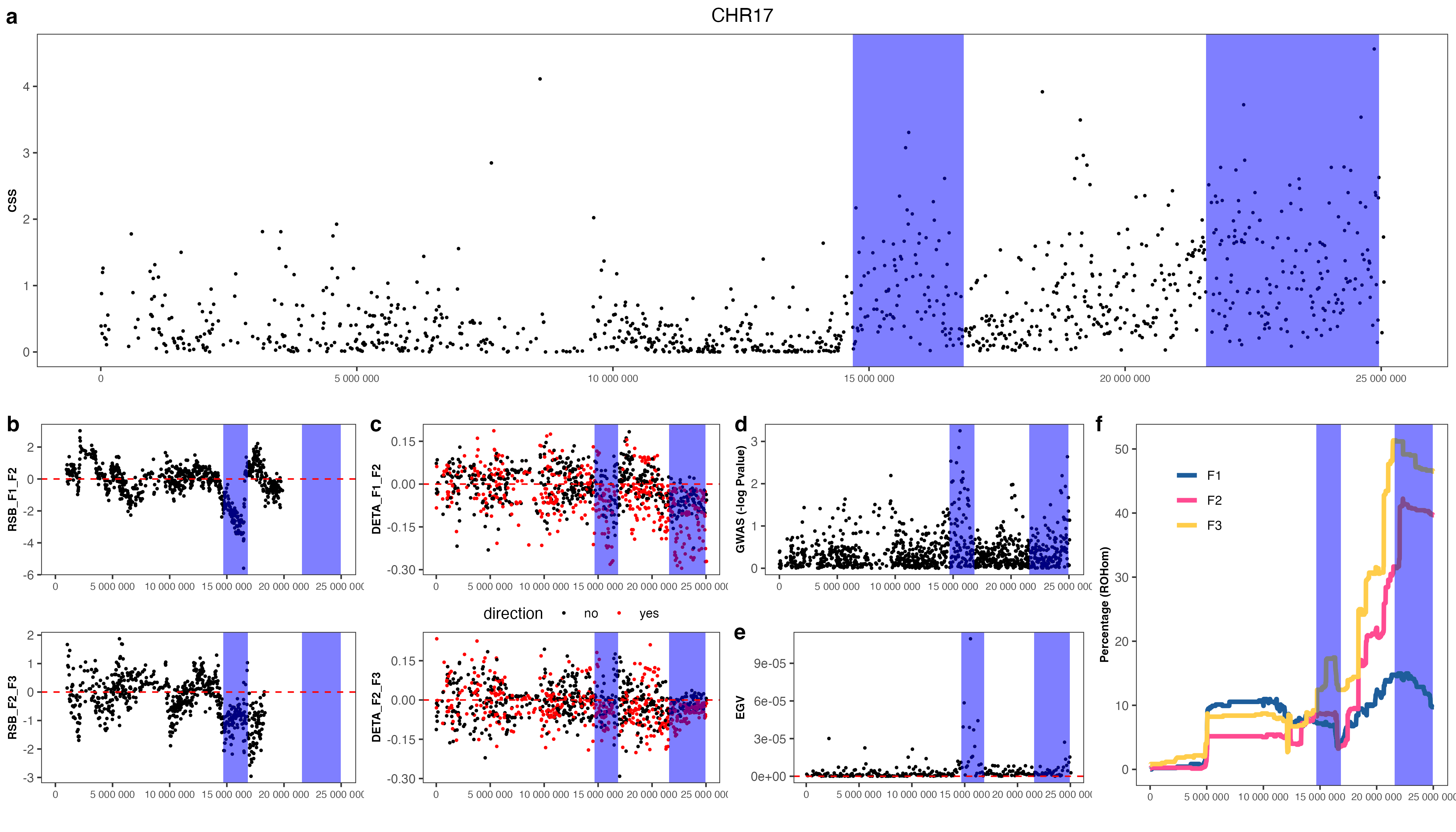

Supplement: Supplementary file 4 — FIGURE S4. Detailed of PSR on chromosome 17. [file EVA-18-e70120-s007.png]

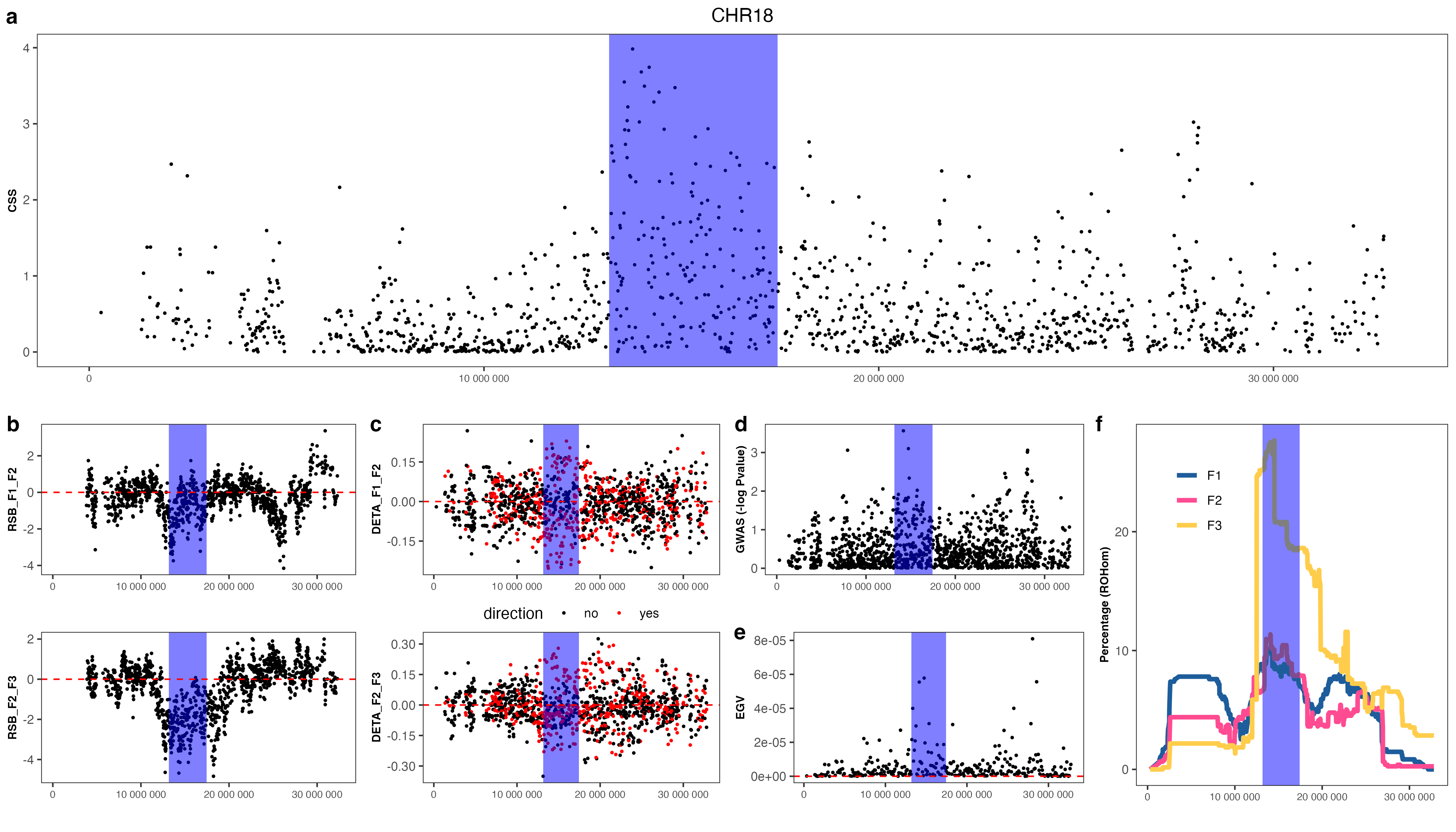

Supplement: Supplementary file 5 — FIGURE S5. Detailed of PSR on chromosome 18. [file EVA-18-e70120-s004.png]

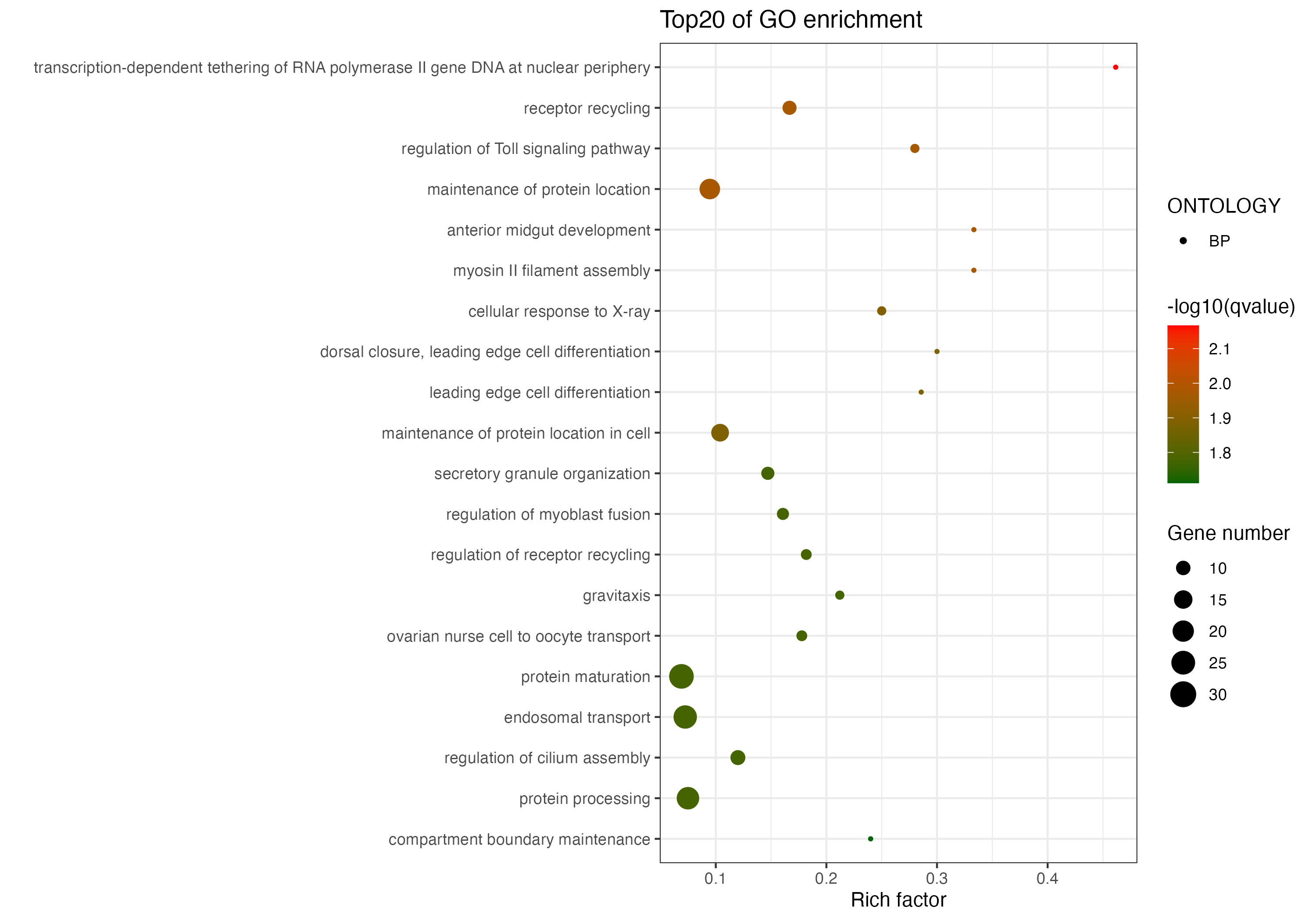

Supplement: Supplementary file 6 — FIGURE S6. The top 20 enriched GO terms for all of genes involved in the PSRs. [file EVA-18-e70120-s003.png]

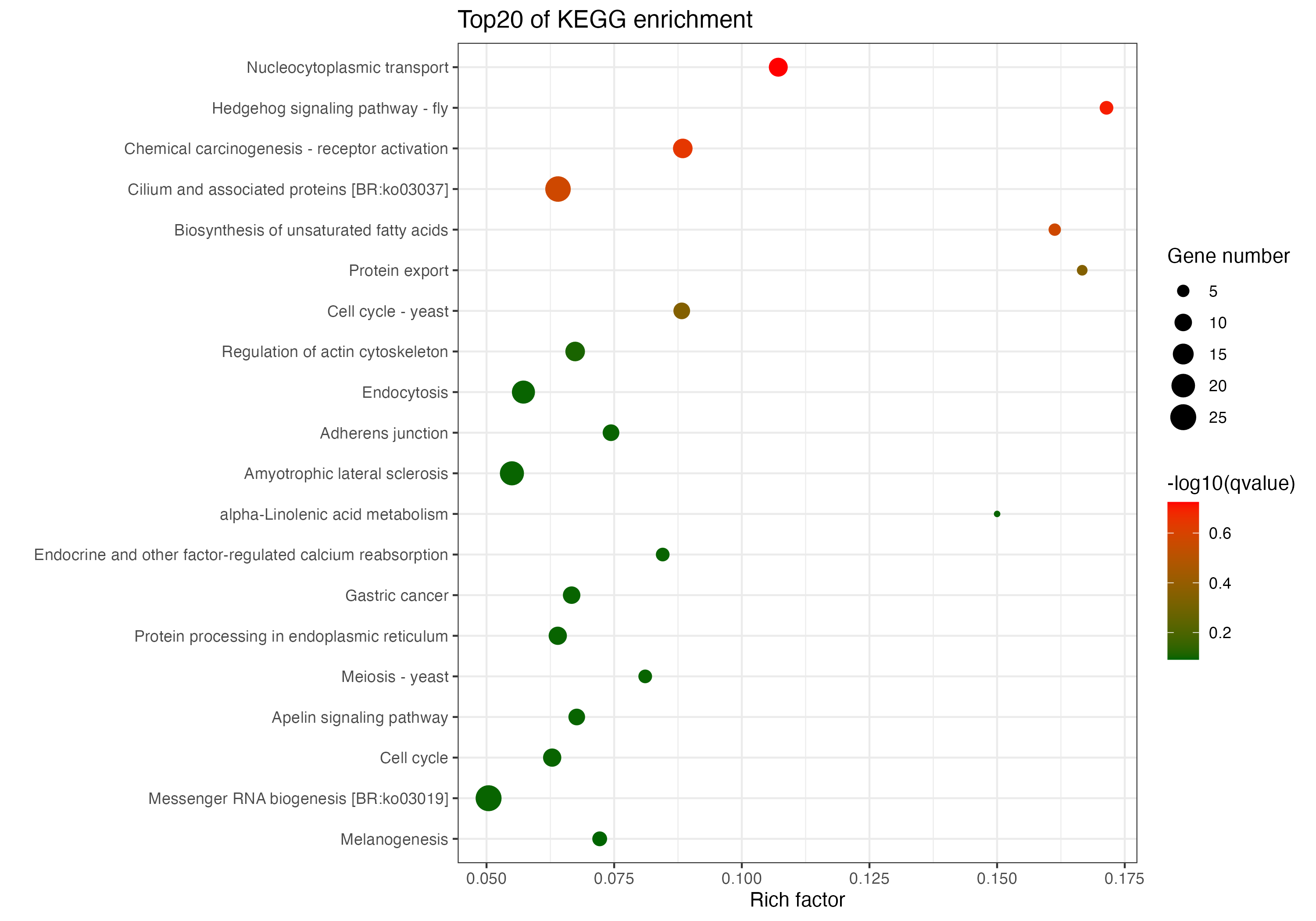

Supplement: Supplementary file 7 — FIGURE S7. The top 20 enriched KEGG pathways for all of genes involved in the PSRs. [file EVA-18-e70120-s009.png]
